# Branchial cleft carcinoma with cervical lymph node metastasis: a Case Report

**DOI:** 10.3389/fonc.2025.1639480

**Published:** 2025-11-21

**Authors:** Xiangyu Diao, Xuan Han, Hongwei Zuo, Fenglin Sun

**Affiliations:** 1 Department of Throat, Head and Neck Surgery, Zibo Central Hospital, Zibo, China; 2 Department of Plastic and Aesthetic Surgery, Zibo Central Hospital, Zibo, China

**Keywords:** branchial cleft carcinoma, lymph node metastasis, cervical lymph node dissection, radiotherapy, follow-up

## Abstract

Branchial cleft carcinoma is a rare malignant tumor originating from the epithelium of branchial cleft cysts, which are highly susceptible to misdiagnosis. There is a lack of systematic consensus on its lymph node metastasis pattern and standardized treatment protocol. We report a case of branchial cleft carcinoma with cervical lymph node metastasis and discuss its diagnostic criteria, clinicopathological features, lymph node metastatic characteristics, and treatment strategies. This case report supports the notion that branchial cleft carcinoma should be diagnosed according to Khafif’s criteria, and long-term follow-up is necessary to reduce the misdiagnosis rate. And treatment should be based on radical tumor resection combined with ipsilateral cervical lymph node dissection, and adjuvant radiotherapy is recommended for the presence of lymph node metastasis or locally invasive growth.

## 1 Introduction

Branchial cleft carcinoma is a malignant tumor originating from embryonic branchial cleft remnants or branchial cleft cystic epithelium. The first case of branchial cleft carcinoma was reported by Volkmann (1) in 1882, but due to the extremely low incidence of the disease, many scholars believed that branchial cleft carcinoma did not exist, but rather originated from the cystic metastatic lymph nodes of the oropharyngeal and nasopharyngeal occult primary cancers. It was not until Martin et al. (2) in 1950 and Khafif et al. (3) in 1989, successively proposed strict diagnostic criteria, that the diagnosis of branchial cleft carcinoma gradually reached a consensus. However, at present, the disease is mostly limited to individual case reports, and there is a lack of a summary of its lymph node metastasis pattern, surgical scope, and indications for adjuvant radiotherapy. In this paper, we would like to analyze the clinical data of a case of branchial cleft carcinoma with delayed cervical lymph node metastasis admitted to our hospital with a follow-up period of up to 5 years, and discuss the following key issues in conjunction with the relevant international literature: (1) the key points of diagnosis of branchial cleft carcinoma and its lymph node metastasis; (2) the pattern of lymph node metastasis of branchial cleft carcinoma; (3) the selection of the treatment plan, including the scope of surgery and the indications for adjuvant radiotherapy; and (4) the significance of long-term follow-up. The above discussion aims to provide a possible experience for the clinical diagnosis and treatment of branchial cleft carcinoma.

## 2 Case presentation

The patient, male, 67 years old, presented to the hospital on 8 June 2020 with the chief complaint of ‘discovery of left submandibular swelling for more than 10 days’. The patient complained of an unintentional discovery of a left submandibular mass 10 days ago, with no fever, chills, pain, hoarseness, dyspnea, dysphagia, or other discomforts. Physical examination: a hard mass of about 3.5cm in diameter was detected in the left submandibular region, with no compression pain, clear borders and good mobility, no redness or swelling of the skin on the surface, and a normal skin temperature. Enhanced CT of the neck showed a 2.7×2.2 cm cystic mass in the space between the left submandibular gland and the sternocleidomastoid muscle, with multiple internal segregations and no enhancement of the cystic part. No significant space-occupying lesions were identified in the nasopharynx, oropharynx, hypopharynx, larynx, or bilateral parapharyngeal spaces, and no abnormal changes in morphology or density were observed in the thyroid gland, bilateral parotid glands, or submandibular glands. Electronic nasopharyngeal laryngoscopy revealed no abnormalities in the nasopharynx, oropharynx, hypopharynx, or larynx. Color Doppler ultrasound of the thyroid and submandibular glands, along with chest CT, showed no significant abnormalities. After perfecting the preoperative preparation, the mass resection was performed on 11 June 2020 under general anesthesia. Intraoperatively, the mass was located between the deep surface of the upper 1/3 of the anterior border of the left sternocleidomastoid muscle and the superficial surface of the cervical sheath. The tumor was cystic, about 3.5×2.5×2.5 cm in size, with intact peritoneum and no obvious adhesion to the surrounding tissues. There were no obvious enlarged lymph nodes around the mass. Postoperative pathology showed that the branchial cleft cyst was partially malignant as a highly-moderately differentiated squamous cell carcinoma, accompanied by local necrosis and hemorrhage, and no cancer cells were seen at the margin. Immunohistochemistry showed: CKAE1/AE3 (+); CK5/6 (+); P63 (+); P40 partially (+); Vimentin (-); CK7 (-); CK20 (-); GATA-3 (-); TTF-1 (-); P16 (-); P53 (-); Ki-67: (+) S accounted for 70% of the total; and the value of EB-DNA was 5.08E + 002. Following surgery, our hospital convened a multidisciplinary team (MDT) consultation. Based on the Khafif diagnostic criteria, this case was diagnosed as branchial cleft carcinoma. The MDT committee decided against adjuvant radiotherapy for the time being, based on negative pathological margins, clinically negative lymph nodes, and the absence of other primary lesions identified during comprehensive examinations. Considering the possibility of cervical metastasis from an unknown primary, the patient was instructed to undergo comprehensive examinations every six months. These include electronic nasopharyngeal laryngoscopy (with particular focus on the Waldeyer’s ring region), neck and chest CT scans, neck color Doppler ultrasound, and a full physical examination.

**Figure 1:**
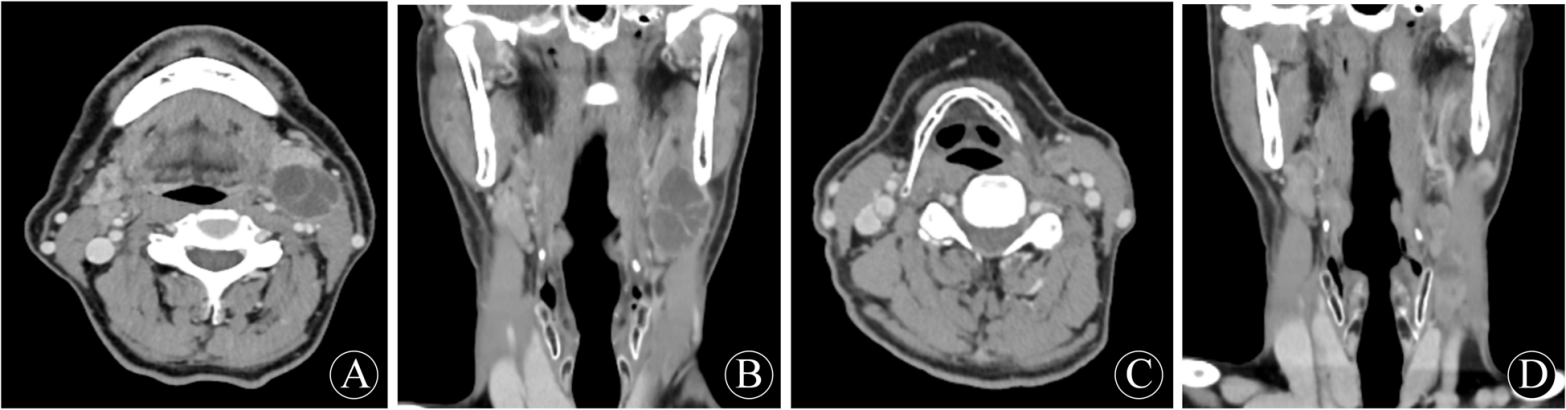
First preoperative neck enhancement CT performance: **(A)** transverse position; **(B)** coronal position. Second preoperative neck enhancement CT performance: **(C)** transverse position; **(D)** coronal position.

**Figure 2:**
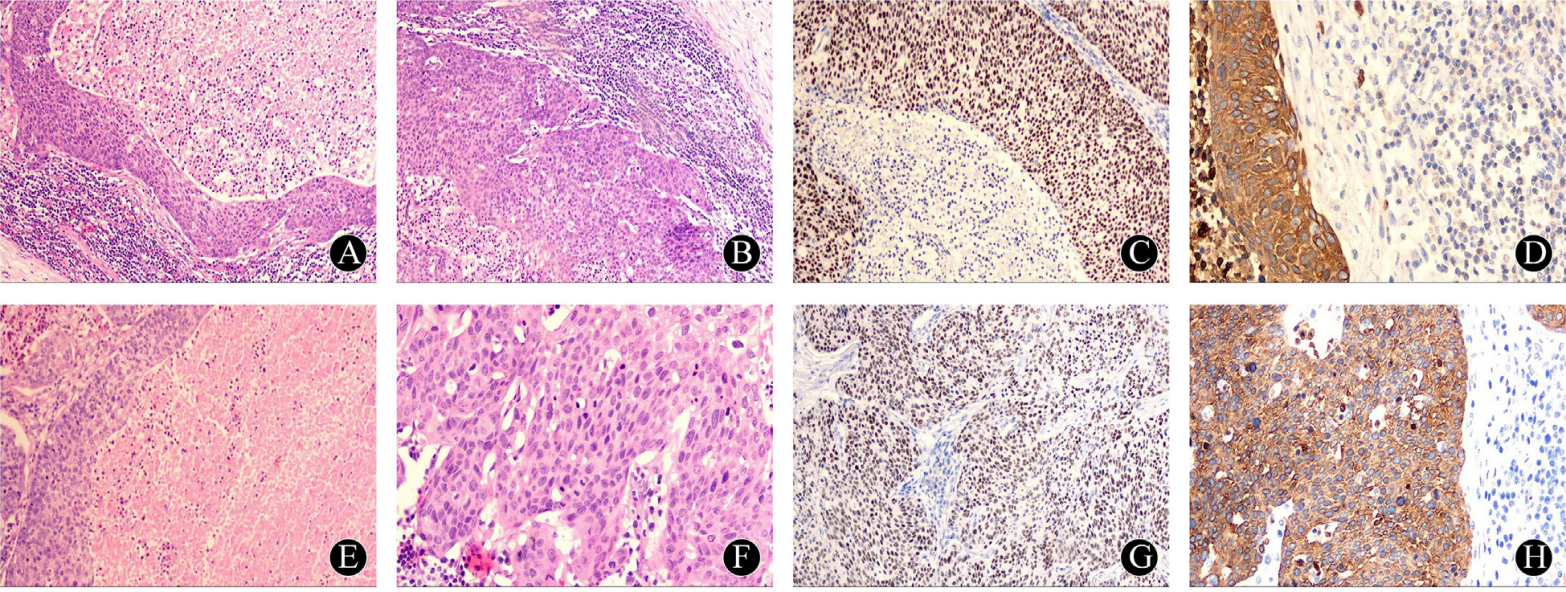
Pathology and immunohistochemistry after the first operation: **(A)** The squamous epithelial cells of the capsule wall gradually transitioned from atypical hyperplasia to squamous cell carcinoma, HE×40; **(B)** The capsule wall epithelium was arranged in a complex layer, with obvious intercellular bridges, extracapsular lymphocytes and lymphoid follicles visible, HE×100 times; **(C)** Immunohistochemistry: P63 cell nuclei diffusely positive, HE×100; **(D)** Immunohistochemistry: CKAE1/AE3 cell membrane positive, HE×400. Pathology and immunohistochemistry after the first operation: **(E)** large number of necrotic and apoptotic cells were seen in the cystic lumen, HE×100; **(F)** large number of pathologic nuclear divisions were seen in the cellular nests, HE×400; **(G)** Immunohistochemistry: P63 cell nuclei were diffusely positive, HE×100; **(H)** Immunohistochemistry: CK5/6 cell membrane positive, HE×400.

On 21 January 2025, the patient was readmitted to the hospital with a left cervical mass that had been present for more than 20 days. He complained of a left-sided neck swelling found after an upper respiratory tract infection more than 20 days ago. Accompanied by fever, sore throat, neck swelling, and pain, no symptoms such as limited neck movement, choking on food, or dyspnea. He was treated with antibiotics on his own, but the swelling did not subside. Physical examination: a round-like mass was palpated at the anterior border of the lower middle part of the left sternocleidomastoid muscle, about 3.0 cm×1.5 cm in size, tough, with poorly defined borders, with acceptable mobility and mild pressure pain. Enhanced CT of the neck showed multiple round-like nodules at the anterior and medial border of the left sternocleidomastoid muscle, about 3.1×1.5×1.2cm in size, and the enhanced lesions showed obvious uneven enhancement, which was considered to be enlarged lymph nodes in the left cervical level II and III. No significant lymph node enlargement was observed in the right neck. No significant space-occupying lesions were identified in the nasopharynx, oropharynx, hypopharynx, or parapharyngeal spaces. No abnormalities were noted in the thyroid, parotid, or submandibular glands. Electronic nasopharyngeal laryngoscopy and chest CT revealed no significant abnormalities. According to the previous history of malignant cyst of the left neck branchial cleft, metastatic lymph node cancer from branchial cleft carcinoma could not be excluded. After perfect preoperative preparation, left elective cervical lymph node dissection (level II-V) was performed under general anesthesia. During the operation, several enlarged lymph nodes were found, which were mainly located in the left cervical levels II and III. One of the larger lymph nodes in level II was partially adherent to the inferior border of the parotid gland. A tough lymph node of about 3.0×1.5 cm in level III was adherent to the internal jugular vein. In the fatty tissue between the left sternocleidomastoid muscle and the platysma muscle, there were two tough lymph nodes with a diameter of about 0.5 cm, which were recorded as superficial cervical lymph nodes. Postoperative pathology showed: metastatic carcinoma in lymph nodes (10/25), including level II (3/8), level III (3/7), level IV (2/7), level V (0/1), and the superficial cervical region (2/2). Immunohistochemistry: CKAE1/AE3: (+); CK5/6: (+); P63: (+); Vimentin (-); P16: (-); CK7: (-); Ki-67: (+) S was 60%. The diagnosis of branchial cleft carcinoma with cervical lymph node metastasis was made based on two pathological and immunohistochemical findings and history. The patient did not experience any postoperative complications corresponding to vascular or nerve injury. Four weeks after surgery, following the recommendation of the MDT committee, the patient underwent adjuvant radiotherapy (covering the left neck II-V regions and Waldeyer’s ring area, with a total dose of 60 Gy in 30 fractions). On review 4 months after surgery, no tumor recurrence, metastasis, or other primary cancers were seen on systemic examination. The examination results are shown in **Figure 1** and **Figure 2**.

## 3 Discussion

Branchial cleft cysts are congenital branchial cleft anomalies formed by embryonic branchial cleft remnant tissue. Theoretically, malignant tumors originating from the epithelial tissue of embryonic branchial cleft remnants or branchial cleft cysts can be called primary branchial cleft carcinomas, but these tumors are rare in clinical practice. The diagnosis of primary branchial cleft carcinoma has only been taken seriously since Volkmann (1) first reported it in 1882. However, due to the lack of diagnostic criteria, more than 250 cases of primary branchial cleft carcinoma reported between 1882 and 1950 were confirmed to be misdiagnosed in subsequent studies (2). Therefore, the existence of primary branchial cleft carcinoma has been controversial. It was not until 1950 that Martin et al. (2) formulated the first strict diagnostic criteria for branchial cleft carcinoma in terms of site of occurrence, histopathology, and differential diagnosis. It included the following four items: ①the mass was located at the anterior border of the sternocleidomastoid muscle, from the ear screen to the clavicle; ② there was a normal branchial cleft epithelium on histopathology; ③ the patient was followed up for more than 5 years and no other primary tumor had been found; ④ there was histological evidence of carcinoma within the branchial cleft cyst epithelium. However, the 5-year follow-up proposed by Martin et al. is not the gold standard, and Thompson et al. (4) reported a case in which the presence of other primary cancers was found 11 years after surgery. Therefore, in 1989, Khafif et al. (3) modified Martin’s diagnostic criteria, including: ① the anatomical location of the tumor is consistent with branchial cleft cyst or branchial cleft fistula; ② the histological manifestation is consistent with the remains of branchial cleft; ③ there is carcinoma within the wall of the epithelial cyst; ④ there is a transition zone from normal squamous epithelium to squamous cell carcinoma; ⑤ there is no primary carcinoma elsewhere after investigation. Most scholars have recognized Khafif’s diagnostic criteria, which are still in use today. This case met the diagnostic criteria for branchial cleft carcinoma: (1) The site of tumor occurrence met Khafif diagnostic criteria ①; (2) Two pathologists were requested to re-examine the pathology slides. In the first postoperative pathology slides, squamous carcinoma cells were observed on the normal cystic epithelium, and normal squamous epithelial cells were seen gradually transitioning to carcinoma cells, which met Khafif’s diagnostic criteria ②, ③ and ④; (3) After the onset of the disease, the patient underwent several nasopharyngolaryngoscopic and cervico-thoracic imaging examinations which did not reveal any tumor of other origins. This fulfilled Khafif’s diagnostic criteria ⑤; (4) The patient’s initial discovery of the neck mass and the final follow-up examination lasted for 5 years (60 months), and there was no other primary carcinoma, which fulfilled the criterion of Martin (③). In conclusion, this case can be diagnosed as branchial cleft carcinoma.

Whether the metastatic lymph nodes in this case originated from branchial cleft carcinoma needs to be analyzed from multiple perspectives: (1) Pathological and immunohistochemical perspectives: the branchial cleft carcinoma in this case was highly-moderately differentiated squamous cell carcinoma, which was of the same pathological type as the metastatic lymph nodes, with the same degree of differentiation. The specific markers CKAE1/AE3, CK5/6, and P63 were all (+), Vimentin and CK7 were all (-), and Ki-67 expression was similar in both immunohistochemistry, which was consistent with the characteristics of the same clonal origin. (2) Anatomical perspective: the location of this branchial cleft carcinoma and the distribution of metastatic lymph nodes were consistent with the lymphatic drainage pathway. (3) Clinical and imaging perspective: no other primary cancers were found on physical examination, electronic nasopharyngolaryngoscopy, ultrasound, and CT, and there was no evidence related to other primary cancers in 5 years of follow-up, so other primary cancers could be excluded. In conclusion, through the comprehensive analysis of pathological consistency, anatomical reasonableness, and clinical exclusivity, it can be strongly proven that the metastatic lymph node originated from branchial cleft carcinoma in this case.

Regarding lymph node metastasis of branchial cleft carcinoma, most of the published international academic literature is limited to individual case reports. There is a lack of a comprehensive and systematic summary of the metastatic characteristics. However, the characteristics of lymph node metastasis play a decisive role in the choice of treatment plan for branchial cleft carcinoma, which directly affects the therapeutic effect and prognosis of this disease. In this case, the metastatic lymph nodes were mainly concentrated in level II (3/8) and level III (3/7) of the ipsilateral neck, which was consistent with the lymphatic drainage of the second branchial cleft. In addition, lymph node metastasis in level IV (2/7) and superficial cervical region (2/2) suggested that the tumor cells might have broken through the local lymphatic barrier, whereas the absence of metastasis in level V (0/1) might be related to the anatomical limitation of branchial cleft carcinoma. The lymph node metastasis rate in this case was 40% (10/25), and the lymph nodes in level II were adherent to the inferior border of the parotid gland, and those in level III were adherent to the internal jugular vein, which reflected the invasiveness of the metastatic foci. This correlates with the invasive features of ‘local necrosis and hemorrhage’ seen in the sections of the primary foci. In terms of molecular pathology, the expression of Ki-67 was 70% in the primary foci and 60% in the metastatic foci, and the high expression of Ki-67 also indicated that the tumor was highly invasive and predicted a high risk of recurrence (5).

The main treatment modality for branchial cleft carcinoma is surgery (6–8), which includes tumor resection and lymph node dissection. For patients with branchial cleft carcinoma in the presence of clinically lymph node-positive (cN+), most scholars agree that complete tumor resection should be accompanied by cervical lymph node dissection on the ipsilateral side (7, 9, 10). However, the question of whether prophylactic cervical lymph node dissection is necessary for patients with clinically lymph node-negative (cN-) branchial cleft carcinoma remains controversial. A study (11) statistically analyzed 32 patients with branchial cleft carcinoma meeting Khafif’s diagnostic criteria, of which 31 patients showed cN- preoperatively, but surprisingly, the overall postoperative lymph node metastasis rate was as high as 25.0%. In the case of the present patient, for example, his imaging findings showed cN- at the time of the first consultation, but metastasis to the ipsilateral cervical lymph node was detected at 4.5 years postoperatively (10/25). Taken together, the above studies and the data from this case suggest that cervical lymph node dissection of the ipsilateral side should be performed along with complete resection of the tumor in patients with branchial cleft carcinoma, regardless of their preoperative clinical lymph node status of cN+ or cN-. However, there are limited studies on the type of cervical lymph node dissection to be performed. Whether radical neck dissection, modified radical neck dissection, or elective neck dissection is more reasonable is still inconclusive and needs to be further investigated. Based on the experience accumulated in this case, the author suggests that patients with branchial cleft carcinoma should undergo at least an elective neck dissection (level II-IV) on the ipsilateral side to effectively reduce the risk of tumor residue and recurrence. When the primary foci or metastatic foci present highly invasive characteristics (such as local tissue infiltration, tumor hemorrhage or necrosis or cystic degeneration, neurovascular invasion, etc.), to remove the potential metastatic foci more thoroughly, the choice of modified radical neck dissection, or even radical neck dissection can be further considered to strive for a more ideal therapeutic effect and prognosis for the patients. Regarding the adjuvant treatment of branchial cleft carcinoma, Bhuvanesh et al. (12) pointed out that radiotherapy could be withheld if no lymph node metastasis was found on pathological examination after tumor resection and ipsilateral cervical lymph node dissection. However, in cases with locally invasive growth or lymph node metastasis, it is generally accepted that postoperative adjuvant radiotherapy helps prevent recurrence of branchial cleft carcinoma (7, 9, 10, 13). And the radiation field should include the ipsilateral neck and Waldeyer’s ring area to kill other occult primary foci that may exist in this area (14). For patients with poor differentiation, rapid progression, and no indication for surgery, a combined treatment plan of palliative surgery combined with radiotherapy may be considered (7, 10, 13).

In addition, cystic metastatic squamous carcinoma of the neck is often misdiagnosed as branchial cleft carcinoma in clinical practice, especially when its primary cancer is occult. The main reasons for this type of misdiagnosis (15) are (1) The jugulodigastric lymph node are in the same location as most branchial cleft cysts; (2) the incidence of metastatic squamous cell carcinoma is much higher than that of branchial cleft carcinoma; (3) metastatic carcinoma of the neck may be used as the first symptom of occult carcinoma of upper respiratory and gastrointestinal tracts, etc.; and (4) it is difficult to differentiate cystic degeneration between branchial cleft carcinoma and metastatic squamous cell carcinoma histologically. Currently, international reports in the literature point out that most of these cystic lymph nodes originate from squamous carcinomas of Waldeyer’s ring (tonsil, tongue root, nasopharynx, etc.) and are associated with HPV infection, with cancers of the tonsil, tongue root, and other oropharyngeal cancers being the most common (4, 16–18). There are also case reports about nasopharyngeal carcinoma, laryngeal carcinoma, hypopharyngeal carcinoma, cystic lymph node metastasis of papillary thyroid carcinoma, and ectopic papillary thyroid carcinoma that were misdiagnosed as branchial cleft carcinoma (19–22). Thompson et al. (4) reported 87 cases of misdiagnosis out of 136 patients, and the time between misdiagnosis of branchial cleft carcinoma and detection of their primary cancer was shorter than 1 month and longer than 11 years, with an average of 12.4 months. In the four cases of misdiagnosed patients reported by Huang et al. (23), two cases were found to have other primary cancers during treatment, one case was found 4 months after surgery, and one case was found 41 months after surgery. In the 15 misdiagnosed patients reported by Liu et al. (20), their primary cancers were found in the shortest time of 4 days, the longest time of 76 months, and the average time of 12 months. Therefore, for patients who have been diagnosed with branchial cleft carcinoma, the search for other possible primary cancers should still not be easily abandoned. Through questioning, physical examination, electronic nasopharyngeal laryngoscopy, gastroenteroscopy, ultrasound, CT, MRI, and positron emission tomography/computed tomography (PET-CT), focusing on the oropharynx, nasopharynx, hypopharynx, and thyroid, we can further rule out other primary cancers and reduce the possibility of missed diagnosis. It can also effectively monitor the recurrence and metastasis of branchial cleft carcinoma, creating opportunities for early treatment. Therefore, follow-up should be carried out in a long-term, regular, standardized, and effective manner.

Furthermore, the NCCN Head and Neck Cancer Guidelines recommend that patients with metastatic neck disease of occult primary undergo bronchoscopy, palatine tonsillectomy, and tongue base mucosal biopsy under general anesthesia (24). These procedures were not performed in this patient due to his refusal of invasive procedures. However, during our 5-year (60-month) follow-up, rigorous monitoring of the nasopharynx, oropharynx (tonsils, tongue base), larynx, hypopharynx, and neck/chest regions was conducted through repeated electronic nasopharyngeal laryngoscopy (particularly of the Waldeyer’s ring area), neck/chest CT, neck ultrasound, and comprehensive physical examinations. No additional lesions were detected. Statistical studies indicate that the average time to identify the primary lesion in cases misdiagnosed as branchial cleft carcinoma is approximately 12 months (4, 20). This case surpasses current research standards in both follow-up duration and monitoring scope. Combined with full compliance with Martin and Khafif diagnostic criteria (2, 3), the diagnosis of branchial cleft carcinoma in this patient is reliable. Nevertheless, the authors recommend that, when feasible, future studies should actively incorporate bronchoscopy, tonsillectomy, and tongue base mucosal biopsy to enhance diagnostic rigor and reduce the potential for overlooking primary tumors.

## Data Availability

The original contributions presented in the study are included in the article/supplementary material. Further inquiries can be directed to the corresponding author.

## References

[1] Volkmann R . Das tiefe branchiogene Halskarcinom. Zentralbl Chir. (1882) 1:49–51.

[2] Martin H Morfit HM Ehrlich H . The case for branchiogenic cancer (malignant branchioma). Ann Surg. (1950) 132:867–87. doi: 10.1097/00000658-195011000-00002, PMID: 14771797 PMC1616605

[3] Khafif RA Prichep R Minkowitz S . Primary branchiogenic carcinoma. Head Neck. (1989) 11:153–63. doi: 10.1002/hed.2880110209, PMID: 2656582

[4] Thompson LD Heffner DK . The clinical importance of cystic squamous cell carcinomas in the neck: a study of 136 cases. Cancer. (1998) 82:944–56. doi: 10.1002/(SICI)1097-0142(19980301)82:5<944::AID-CNCR21>3.0.CO;2-#, PMID: 9486586

[5] Scholzen T Gerdes J . The Ki-67 protein: from the known and the unknown. J Cell Physiol. (2000) 182:311–22. doi: 10.1002/(SICI)1097-4652(200003)182:3<311::AID-JCP1>3.0.CO;2-9, PMID: 10653597

[6] Briggs RD Pou AM Schnadig. VJ . Cystic metastasis versus branchial cleft carcinoma: a diagnostic challenge. Laryngoscope. (2002) 112:1010–4. doi: 10.1097/00005537-200206000-00014, PMID: 12160265

[7] Zeng B Li W Yang L Kong. W . Branchiogenic carcinoma in the parotid gland. Chin Med J (Engl). (2019) 132:2388–9. doi: 10.1097/CM9.0000000000000451, PMID: 31567380 PMC6819045

[8] Gao R Xiao Y Gao J . Diagnosis and treatment of bilateral primary branchiogenic carcinoma: A case of Malignant transformation of branchial cleft cyst. J Stomatol Oral Maxillofac Surg. (2025) 126:102028. doi: 10.1016/j.jormas.2024.102028, PMID: 39226985

[9] Devaney KO Rinaldo A Ferlito A Silver CE Fagan JJ Bradley PJ . Squamous carcinoma arising in a branchial cleft cyst: have you ever treated one? Will you? J Laryngol Otol. (2008) 122:547–50. doi: 10.1017/S0022215107001004, PMID: 18005502

[10] Katori H Nozawa A Tsukuda M . Post-operative adjuvant chemoradiotherapy with carboplatin and 5-fluorouracil for primary branchiogenic carcinoma. J Laryngol Otol. (2005) 119:467–9. doi: 10.1258/0022215054273241, PMID: 15992474

[11] Ye P Wei T Yang H Peng Q Cai Y . Treatment and prognosis of branchiogenic carcinoma: a systematic analysis. J Shanxi Med Univ. (2018) 49:1112–6. doi: 10.13753/j.issn.1007-6611.2018.09.022

[12] Singh B Balwally AN Sundaram K Har-El G Krgin. B . Branchial cleft cyst carcinoma: myth or reality? Ann Otol Rhinol Laryngol. (1998) 107:519–24. doi: 10.1177/000348949810700611, PMID: 9635463

[13] Sprave T Ruhle A Hees K Kalckreuth T Verma V Stoian R . Radiotherapeutic management of cervical lymph node metastases from an unknown primary site - experiences from a large cohort treated with modern radiation techniques. Radiat Oncol. (2020) 15:80. doi: 10.1186/s13014-020-01529-z, PMID: 32293497 PMC7158130

[14] Gourin CG Johnson JT . Incidence of unsuspected metastases in lateral cervical cysts. Laryngoscope. (2000) 110:1637–41. doi: 10.1097/00005537-200010000-00012, PMID: 11037817

[15] Park SS Karmody CS . The first branchial cleft carcinoma. Arch Otolaryngol Head Neck Surg. (1992) 118:969–71. doi: 10.1001/archotol.1992.01880090085022, PMID: 1503725

[16] Goldenberg D Sciubba J Koch WM . Cystic metastasis from head and neck squamous cell cancer: a distinct disease variant? Head Neck. (2006) 28:633–8. doi: 10.1002/hed.20381, PMID: 16477605

[17] Regauer S Mannweiler S Anderhuber W Gotschuli A Berghold A Schachenreiter J . Cystic lymph node metastases of squamous cell carcinoma of Waldeyer’s ring origin. Br J Cancer. (1999) 79:1437–42. doi: 10.1038/sj.bjc.6690229, PMID: 10188887 PMC2362723

[18] Goyal N Zacharia TT Goldenberg D . Differentiation of branchial cleft cysts and Malignant cystic adenopathy of pharyngeal origin. AJR Am J Roentgenol. (2012) 199:W216–221. doi: 10.2214/AJR.11.8120, PMID: 22826424

[19] Ida JB Stark MW Xiang Z Fazekas-May. MM . Laryngeal cancer involving a branchial cleft cyst. Head Neck. (2011) 33:1796–9. doi: 10.1002/hed.21476, PMID: 20629072

[20] Liu Q Luo J . Analysis of cases of cervical cystic lymph node metastasis with an unknown primary misdiagnosed as branchial cleft carcinoma. Chin J Oncol. (2024) 46:583–9. doi: 10.3760/cma.j.cn112152-20231024-00248, PMID: 38880737

[21] Park J Kwon SY Kim NH Baik SH Choi. DS . Papillary thyroid carcinoma arising in a branchial cleft cyst. Thyroid. (2010) 20:347–9. doi: 10.1089/thy.2009.0124, PMID: 20187788

[22] Wang W Ni X Gu Y An R Wang C Zhang. J . Unusual coexistence: branchial cleft cyst harboring papillary thyroid carcinoma with lymph node metastasis-a rare case report and clinical insights. Front Oncol. (2024) 14:1378405. doi: 10.3389/fonc.2024.1378405, PMID: 38665942 PMC11043480

[23] Huang C Wang H Kong Y Wang. J . Metastatic cystic squamous cell carcinoma in the neck mistaken as primary branchial cleft carcinoma: a report of 4 cases. Chin J Oncol. (2004) 26:60–3. doi: 10.3760/j.issn:0253-3766.2004.10.016, PMID: 15634531

[24] National Comprehensive Cancer Network . NCCN clinical practice guidelines in oncology: head and neck cancers. Version 5. (2025). 10.6004/jnccn.2020.0031 32634781

